# (*E*)-4-Bromo-*N*-(2,3,4-trimeth­oxy­benzyl­idene)aniline

**DOI:** 10.1107/S1600536810028163

**Published:** 2010-07-21

**Authors:** Karla Fejfarová, Aliakbar Dehno Khalaji, Michal Dušek

**Affiliations:** a Institute of Physics, Na Slovance 2, 182 21 Praha 8, Czech Republic; b Department of Chemistry, Faculty of Science, Golestan University, Gorgan, Iran

## Abstract

The title Schiff base compound, C_16_H_16_BrNO_3_, adopts an *E* configuration with respect to the C=N bond. The dihedral angle between the two aromatic rings is 64.02 (6)°.

## Related literature

For applications of Schiff-base compounds, see: Yildiz *et al.* (2008 ▶); Hijji *et al.* (2009 ▶); Karakas *et al.* (2008 ▶); Hadjoudis *et al.* (2004 ▶). For related structures, see: Khalaji *et al.* (2007 ▶, 2008 ▶, 2009 ▶, 2010 ▶); Khalaji & Harrison (2008 ▶); Khalaji & Simpson (2009 ▶). For standard bond lengths, see: Allen *et al.* (1987 ▶).
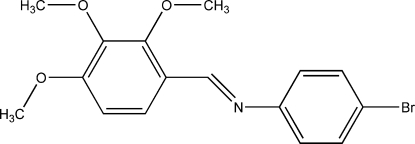


## Experimental

### 

#### Crystal data

C_16_H_16_BrNO_3_
*M* *_r_* = 350.2
Triclinic, 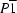

*a* = 7.9103 (3) Å
*b* = 9.9902 (4) Å
*c* = 10.7821 (3) Å
α = 93.068 (3)°
β = 108.568 (3)°
γ = 109.679 (3)°
*V* = 748.10 (5) Å^3^
*Z* = 2
Cu *K*α radiation
μ = 3.83 mm^−1^
*T* = 120 K
0.49 × 0.38 × 0.25 mm

#### Data collection

Oxford Diffraction Xcalibur diffractometer with an Atlas (Gemini Ultra Cu) detector
Absorption correction: analytical (*CrysAlis PRO*; Oxford Diffraction, 2009 ▶) *T*_min_ = 0.308, *T*_max_ = 0.631
11571 measured reflections
2546 independent reflections
2485 reflections with *I* > 3σ(*I*)
*R*_int_ = 0.023

#### Refinement

*R*[*F* ^2^ > 2σ(*F* ^2^)] = 0.026
*wR*(*F* ^2^) = 0.093
*S* = 1.73
2546 reflections
190 parameters
H-atom parameters constrained
Δρ_max_ = 0.29 e Å^−3^
Δρ_min_ = −0.28 e Å^−3^

### 

Data collection: *CrysAlis PRO* (Oxford Diffraction, 2009 ▶); cell refinement: *CrysAlis PRO*; data reduction: *CrysAlis PRO*; program(s) used to solve structure: *SIR2002* (Burla *et al.*, 2003 ▶); program(s) used to refine structure: *JANA2006* (Petříček *et al.*, 2006 ▶); molecular graphics: *DIAMOND* (Brandenburg & Putz, 2005 ▶); software used to prepare material for publication: *JANA2006*.

## Supplementary Material

Crystal structure: contains datablocks global, I. DOI: 10.1107/S1600536810028163/fk2021sup1.cif

Structure factors: contains datablocks I. DOI: 10.1107/S1600536810028163/fk2021Isup2.hkl

Additional supplementary materials:  crystallographic information; 3D view; checkCIF report

## Figures and Tables

**Fig. 1.**
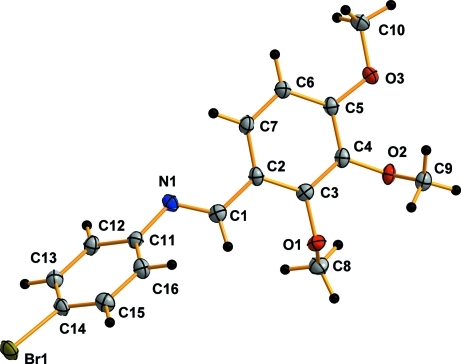
The molecular structure of the title compound with atom-labeling scheme. Displacement ellipsoids are drawn at the 50% probability level.

## supplementary crystallographic information

### Comment

The chemistry of Schiff-bases is very diverse because of a variety of possible substituents with different electron donating and withdrawing groups (Yildiz *et al.*, 2008; Hijji *et al.*, 2009; Karakas *et al.*, 2008). These compounds have been studied for their use as anion sensors (Hijji *et al.*, 2009), antimicrobial activity (Yildiz *et al.*, 2008), photochromism and thermochromism (Hadjoudis *et al.*, 2004) and nonlinear optical properties (Karakas *et al.*, 2008). As a continuation of our work on the synthesis and structural characterization of Schiff-base compounds we report the synthesis and crystal structure of (*E*)-4-bromo-*N*-(2,3-dimethoxybenzylidene)aniline (1).

An *ORTEP* plot, with the atomic numbering scheme is depicted in Fig. 1. Bond lengths in the title compound are normal (Allen *et al.*, 1987). The C1—N1 and C11—N1 bond lengths of 1.286 (3) and 1.415 (3) Å, respectively, conform to the value for a double and single bonds as found in similar Schiff-base compounds (Khalaji *et al.*, 2007; Khalaji & Harrison, 2008; Khalaji *et al.*, 2008; Khalaji & Simpson, 2009; Khalaji *et al.*, 2009; Khalaji *et al.*, 2010). The dihedral angle between the two aromatic rings is 64.02 (6)°, while the plane through the central C1—N1—C1—C2 system is inclined at 21.65 (18)° to the dimethoxyphenyl ring and 42.37 (18)° to the bromobenzene ring. The two methoxy groups attached at C3 and C4 are twisted away from the C2—C7 benzene ring, with corresponding torsion angles C8—O1—C3—C2, C9—O2—C4—C3 of 103.6 (2)°, -88.7 (2)°, respectively. The third methoxy group attached at C5 is almost coplanar with the C2—C7 ring, as shown by the torsion angle C10—O3—C5—C6 of -7.2 (3)°.

### Experimental

The title compound was prepared in 83% yield from 2,3,4-trimethoxybenzaldehyde and 4-bromoaniline as reported elsewhere (Khalaji & Harrison, 2008) and recrystallized from chloroform. Anal. Calc. for C_16_H_16_BrNO_3_: C, 54.87; H, 4.60; N, 4.00%. Found: C, 54.66; H, 4.52; N, 4.06%. IR (KBr pellet, cm-1): 2911–2998 (m, C—H aromatic and aliphatic), 2837 (s, –CH=N–); 1615 (s, C=N), 1413–1594 (C=C aromatic).

### Refinement

All hydrogen atoms were discernible in difference Fourier maps and could be refined to reasonable geometry. According to common practice the H atoms were placed in geometrically ideal positions and allowed to ride on their respective parent atoms, with C—H distance of 0.96 Å. The isotropic atomic displacement parameters of hydrogen atoms were evaluated as 1.2**U*_eq_ of the parent atom.

### Crystal data

**Table:**

C_16_H_16_BrNO_3_	*Z* = 2
*M**_r_* = 350.2	*F*(000) = 356
Triclinic, *P*1	*D*_x_ = 1.554 Mg m^−^^3^
Hall symbol: -P 1	Cu *K*α radiation, λ = 1.54184 Å
*a* = 7.9103 (3) Å	Cell parameters from 11782 reflections
*b* = 9.9902 (4) Å	θ = 4.4–66.7°
*c* = 10.7821 (3) Å	µ = 3.83 mm^−^^1^
α = 93.068 (3)°	*T* = 120 K
β = 108.568 (3)°	Irregular shape, colourless
γ = 109.679 (3)°	0.49 × 0.38 × 0.25 mm
*V* = 748.10 (5) Å^3^	

### Data collection

**Table:**

Oxford Diffraction Xcalibur diffractometer with an Atlas (Gemini Ultra Cu) detector	2546 independent reflections
Radiation source: X-ray tube	2485 reflections with *I* > 3σ(*I*)
mirror	*R*_int_ = 0.023
Detector resolution: 10.3784 pixels mm^-1^	θ_max_ = 65.1°, θ_min_ = 4.4°
Rotation method data acquisition using ω scans	*h* = −9→9
Absorption correction: analytical (*CrysAlis PRO*; Oxford Diffraction, 2009)	*k* = −11→11
*T*_min_ = 0.308, *T*_max_ = 0.631	*l* = −12→12
11571 measured reflections	

### Refinement

**Table:**

Refinement on *F*^2^	64 constraints
*R*[*F*^2^ > 2σ(*F*^2^)] = 0.026	H-atom parameters constrained
*wR*(*F*^2^) = 0.093	Weighting scheme based on measured s.u.'s *w* = 1/[σ^2^(*I*) + 0.0025000002*I*^2^]
*S* = 1.73	(Δ/σ)_max_ = 0.010
2546 reflections	Δρ_max_ = 0.29 e Å^−^^3^
190 parameters	Δρ_min_ = −0.28 e Å^−^^3^
0 restraints	

### Special details

**Table:**

Experimental. CrysAlisPro (Oxford Diffraction, 2009). Analytical numeric absorption correction using a multifaceted crystal model.
Refinement. The refinement was carried out against all reflections. The conventional *R*-factor is always based on *F*. The goodness of fit as well as the weighted *R*-factor are based on *F* and *F*^2^ for refinement carried out on *F* and *F*^2^, respectively. The threshold expression is used only for calculating *R*-factors *etc*. and it is not relevant to the choice of reflections for refinement.The program used for refinement, Jana2006, uses the weighting scheme based on the experimental expectations, see _refine_ls_weighting_details, that does not force *S* to be one. Therefore the values of *S* are usually larger than the ones from the *SHELX* program.

### Fractional atomic coordinates and isotropic or equivalent isotropic displacement parameters (Å^2^)

**Table:**

	*x*	*y*	*z*	*U*_iso_*/*U*_eq_	
Br1	0.74999 (3)	0.08307 (2)	0.325568 (16)	0.02973 (14)	
O1	0.27449 (16)	0.17106 (14)	0.96566 (12)	0.0209 (5)	
O2	0.28261 (17)	0.27191 (14)	1.21239 (13)	0.0204 (5)	
O3	0.58807 (17)	0.49203 (14)	1.37866 (12)	0.0217 (5)	
N1	0.7545 (2)	0.28811 (17)	0.86865 (15)	0.0205 (6)	
C1	0.5987 (2)	0.27332 (18)	0.88986 (17)	0.0196 (6)	
C2	0.6019 (2)	0.33388 (19)	1.01757 (17)	0.0183 (6)	
C3	0.4387 (2)	0.27855 (18)	1.05351 (17)	0.0179 (6)	
C4	0.4391 (2)	0.33244 (18)	1.17524 (16)	0.0177 (6)	
C5	0.6034 (2)	0.44399 (19)	1.26324 (17)	0.0188 (6)	
C6	0.7671 (2)	0.50042 (19)	1.22894 (17)	0.0205 (7)	
C7	0.7633 (2)	0.4451 (2)	1.10740 (17)	0.0209 (7)	
C8	0.2463 (3)	0.0301 (2)	1.0001 (2)	0.0275 (7)	
C9	0.1411 (2)	0.3364 (2)	1.16845 (19)	0.0225 (7)	
C10	0.7574 (3)	0.5963 (2)	1.4782 (2)	0.0283 (8)	
C11	0.7401 (2)	0.23365 (19)	0.74029 (17)	0.0190 (6)	
C12	0.8644 (2)	0.16622 (19)	0.73130 (18)	0.0214 (7)	
C13	0.8630 (3)	0.1162 (2)	0.60794 (19)	0.0228 (7)	
C14	0.7397 (3)	0.1383 (2)	0.49439 (18)	0.0212 (7)	
C15	0.6134 (3)	0.2048 (2)	0.50017 (19)	0.0232 (7)	
C16	0.6156 (3)	0.2530 (2)	0.62404 (18)	0.0224 (7)	
H1	0.477112	0.22121	0.820107	0.0236*	
H6	0.880465	0.576586	1.289179	0.0246*	
H7	0.875405	0.484421	1.084082	0.0251*	
H8a	0.117756	−0.035146	0.948638	0.0329*	
H8b	0.336604	−0.004589	0.981472	0.0329*	
H8c	0.265545	0.035921	1.093009	0.0329*	
H9a	0.039226	0.294309	1.201348	0.0269*	
H9b	0.200061	0.438601	1.201714	0.0269*	
H9c	0.089588	0.3194	1.073	0.0269*	
H10a	0.725742	0.625109	1.551587	0.034*	
H10b	0.852651	0.554334	1.508865	0.034*	
H10c	0.807226	0.679308	1.440879	0.034*	
H12	0.95208	0.154077	0.810937	0.0256*	
H13	0.946288	0.067032	0.60173	0.0274*	
H15	0.526458	0.217114	0.42029	0.0278*	
H16	0.530309	0.300348	0.629778	0.0268*	

### Atomic displacement parameters (Å^2^)

**Table:**

	*U*^11^	*U*^22^	*U*^33^	*U*^12^	*U*^13^	*U*^23^
Br1	0.03787 (19)	0.03187 (19)	0.02105 (19)	0.00904 (13)	0.01747 (12)	0.00097 (11)
O1	0.0192 (6)	0.0209 (7)	0.0176 (6)	0.0033 (5)	0.0045 (5)	0.0034 (5)
O2	0.0202 (6)	0.0239 (7)	0.0230 (7)	0.0091 (5)	0.0132 (5)	0.0103 (5)
O3	0.0227 (6)	0.0259 (7)	0.0175 (6)	0.0079 (5)	0.0099 (5)	0.0019 (5)
N1	0.0230 (7)	0.0245 (8)	0.0159 (7)	0.0089 (6)	0.0090 (6)	0.0047 (6)
C1	0.0214 (8)	0.0192 (8)	0.0189 (9)	0.0072 (7)	0.0078 (7)	0.0067 (7)
C2	0.0206 (8)	0.0211 (8)	0.0174 (8)	0.0098 (7)	0.0094 (7)	0.0071 (7)
C3	0.0179 (8)	0.0183 (8)	0.0178 (8)	0.0071 (7)	0.0059 (6)	0.0058 (7)
C4	0.0196 (8)	0.0197 (8)	0.0192 (8)	0.0096 (7)	0.0107 (7)	0.0095 (7)
C5	0.0214 (8)	0.0216 (9)	0.0177 (8)	0.0104 (7)	0.0093 (7)	0.0069 (7)
C6	0.0196 (8)	0.0213 (9)	0.0199 (9)	0.0059 (7)	0.0082 (7)	0.0030 (7)
C7	0.0201 (8)	0.0238 (9)	0.0213 (9)	0.0073 (7)	0.0114 (7)	0.0060 (7)
C8	0.0309 (9)	0.0197 (9)	0.0304 (11)	0.0044 (8)	0.0147 (8)	0.0038 (8)
C9	0.0187 (9)	0.0268 (9)	0.0248 (10)	0.0093 (8)	0.0105 (7)	0.0066 (7)
C10	0.0256 (9)	0.0369 (11)	0.0186 (9)	0.0094 (8)	0.0066 (7)	−0.0024 (8)
C11	0.0192 (8)	0.0185 (8)	0.0188 (9)	0.0039 (7)	0.0091 (7)	0.0044 (7)
C12	0.0208 (8)	0.0256 (9)	0.0208 (9)	0.0097 (7)	0.0098 (7)	0.0078 (7)
C13	0.0232 (9)	0.0227 (9)	0.0265 (10)	0.0097 (7)	0.0126 (7)	0.0055 (7)
C14	0.0261 (9)	0.0205 (9)	0.0186 (9)	0.0053 (7)	0.0140 (7)	0.0022 (7)
C15	0.0277 (9)	0.0249 (9)	0.0185 (9)	0.0105 (8)	0.0088 (7)	0.0083 (7)
C16	0.0255 (9)	0.0241 (9)	0.0223 (9)	0.0118 (7)	0.0116 (7)	0.0066 (7)

### Geometric parameters (Å, °)

**Table:**

Br1—C14	1.908 (2)	C8—H8a	0.96
O1—C3	1.3805 (16)	C8—H8b	0.96
O1—C8	1.438 (2)	C8—H8c	0.96
O2—C4	1.377 (2)	C9—H9a	0.96
O2—C9	1.440 (3)	C9—H9b	0.96
O3—C5	1.365 (2)	C9—H9c	0.96
O3—C10	1.4342 (19)	C10—H10a	0.96
N1—C1	1.286 (3)	C10—H10b	0.96
N1—C11	1.415 (3)	C10—H10c	0.96
C1—C2	1.463 (3)	C11—C12	1.390 (3)
C1—H1	0.96	C11—C16	1.395 (3)
C2—C3	1.408 (3)	C12—C13	1.391 (3)
C2—C7	1.3946 (19)	C12—H12	0.96
C3—C4	1.391 (3)	C13—C14	1.383 (3)
C4—C5	1.3990 (19)	C13—H13	0.96
C5—C6	1.403 (3)	C14—C15	1.387 (3)
C6—C7	1.382 (3)	C15—C16	1.388 (3)
C6—H6	0.96	C15—H15	0.96
C7—H7	0.96	C16—H16	0.96
			
C3—O1—C8	113.46 (13)	O2—C9—H9a	109.4709
C4—O2—C9	112.97 (15)	O2—C9—H9b	109.4713
C5—O3—C10	117.80 (15)	O2—C9—H9c	109.4711
C1—N1—C11	118.43 (13)	H9a—C9—H9b	109.4714
N1—C1—C2	121.70 (13)	H9a—C9—H9c	109.4709
N1—C1—H1	119.1502	H9b—C9—H9c	109.4718
C2—C1—H1	119.1489	O3—C10—H10a	109.4713
C1—C2—C3	119.84 (13)	O3—C10—H10b	109.4716
C1—C2—C7	122.20 (18)	O3—C10—H10c	109.4711
C3—C2—C7	117.96 (17)	H10a—C10—H10b	109.4714
O1—C3—C2	119.60 (16)	H10a—C10—H10c	109.4707
O1—C3—C4	119.38 (16)	H10b—C10—H10c	109.4713
C2—C3—C4	121.01 (13)	N1—C11—C12	117.91 (16)
O2—C4—C3	120.43 (12)	N1—C11—C16	122.8 (2)
O2—C4—C5	119.83 (17)	C12—C11—C16	119.21 (19)
C3—C4—C5	119.66 (17)	C11—C12—C13	120.60 (17)
O3—C5—C4	115.31 (17)	C11—C12—H12	119.6985
O3—C5—C6	124.62 (13)	C13—C12—H12	119.6991
C4—C5—C6	120.06 (17)	C12—C13—C14	118.9 (2)
C5—C6—C7	119.23 (13)	C12—C13—H13	120.5337
C5—C6—H6	120.3876	C14—C13—H13	120.5339
C7—C6—H6	120.3865	Br1—C14—C13	119.31 (18)
C2—C7—C6	122.09 (18)	Br1—C14—C15	118.89 (14)
C2—C7—H7	118.9551	C13—C14—C15	121.8 (2)
C6—C7—H7	118.9554	C14—C15—C16	118.52 (18)
O1—C8—H8a	109.4712	C14—C15—H15	120.7399
O1—C8—H8b	109.4712	C16—C15—H15	120.7397
O1—C8—H8c	109.4713	C11—C16—C15	120.9 (2)
H8a—C8—H8b	109.4713	C11—C16—H16	119.5302
H8a—C8—H8c	109.4709	C15—C16—H16	119.5303
H8b—C8—H8c	109.4714		
			
C8—O1—C3—C2	103.6 (2)	C11—N1—C1—C2	−176.48 (16)
C8—O1—C3—C4	−77.6 (2)	C1—N1—C11—C12	−140.37 (18)
C9—O2—C4—C3	−88.7 (2)	C1—N1—C11—C16	43.7 (3)
C9—O2—C4—C5	94.54 (19)	N1—C1—C2—C3	−159.30 (17)
C10—O3—C5—C4	174.08 (16)	N1—C1—C2—C7	20.0 (3)
C10—O3—C5—C6	−7.2 (3)		

### Hydrogen-bond geometry (Å, °)

**Table:**

*D*—H···*A*	*D*—H	H···*A*	*D*···*A*	*D*—H···*A*
?—?···?	?	?	?	?
